# Shared Decision-Making in the Choice of Renal Replacement Therapy: A Comparative Text Mining Analysis of Physicians and Nurses

**DOI:** 10.3390/nursrep16040142

**Published:** 2026-04-16

**Authors:** Misa Iida, Sumiyo Nabeshima, Sayuri Kaneko, Yuji Kamijo, Toshio Kobayashi, Yukako Ando

**Affiliations:** 1 Graduate School of Nursing, Nagoya City University, Nagoya 467-8601, Japan; iidamisa@med.nagoya-cu.ac.jp; 2 Faculty of Nursing, Kinjo Gakuin University, Nagoya 463-8521, Japan; nabeshima@kinjo-u.ac.jp; 3 Graduate School of Nursing, Miyagi University, Sendai 981-3298, Japan; kanekos@myu.ac.jp; 4 Department of Nephrology, Shinshu University School of Medicine, Matsumoto 390-8621, Japan; yujibeat@shinshu-u.ac.jp; 5 School of Nursing, Hiroshima Bunka Gakuen University, Hiroshima 737-0004, Japan; t-kobayashi@hbg.ac.jp

**Keywords:** renal replacement therapy, shared decision-making, facilitating factors, nurses, physicians, nursing

## Abstract

**Objective:** This study aimed to compare factors facilitating shared decision-making (SDM) in renal replacement therapy decision support between physicians and nurses using text mining analysis. **Methods:** A web-based survey was conducted among 250 physicians and 299 nurses between December 2024 and March 2025. Free-text responses regarding factors facilitating SDM were collected and analyzed using quantitative text analysis. **Results:** Valid responses were obtained from 103 physicians and 122 nurses. Both groups identified six factors, with three shared conceptual domains across physicians and nurses, reflected in three physician factors and four nurse factors. Common domains included “promoting patient and family understanding”, “enhancing staff education”, and “strengthening multidisciplinary collaboration”. Physicians emphasized structural and environmental factors, such as “establishing clinical systems”, “inter-institutional collaboration”, and “securing sufficient time”. In contrast, nurses highlighted practical and interpersonal aspects, including “understanding patients’ values and lifestyles”, “supporting patient-centered decision-making”, and “promoting team-based information sharing”. **Conclusions:** Factors that facilitate SDM in renal replacement therapy include perspectives common to both physicians and nurses, as well as profession-specific perspectives. These findings suggest that integrating organizational support and clinical skills development is crucial for promoting SDM in clinical settings.

## 1. Introduction

Chronic kidney disease (CKD) is a progressive disorder characterized by an irreversible decline in kidney function. Patients who progress to end-stage kidney disease must either choose an available renal replacement therapy such as hemodialysis, peritoneal dialysis, kidney transplantation, or conservative kidney management aimed at symptom control without dialysis or transplantation. Although renal replacement therapy can prolong survival, concerns remain regarding its impact on quality of life, as reflected by the high prevalence of depression following therapy initiation [1,2] and difficulties in maintaining employment [3]. Consequently, decision support incorporating patients’ values and life circumstances has been increasingly emphasized [4,5,6].

In the context of treatment decision-making, shared decision-making (SDM)—a process in which patients and healthcare professionals collaboratively determine treatment options—has attracted attention since the early 2000s [7]. SDM has been shown to improve treatment satisfaction [8,9,10] and promote active patient engagement in care [11], and its use is recommended for supporting decisions related to renal replacement therapy [5]. Furthermore, in a survey of physicians and nurses involved in supporting renal replacement therapy decision-making in Japan, 96.0% reported knowing about SDM [12]. In contrast, a domestic study found that only 22.2% of patients initiating dialysis received SDM-based support [13], suggesting that SDM has not yet been fully established in clinical practice. In addition, as a cultural factor that may hinder the implementation of SDM in Japan, family-centered decision-making rooted in Confucianism, which is characteristic of East Asia, has been noted; this often conflicts with Western notions that emphasize individual autonomy [14].

Previous studies examining physicians’ and nurses’ perspectives on SDM have indicated that interprofessional collaboration and systematic support for decision-making remain insufficient [15]. Therefore, there is still limited understanding of how facilitators of SDM differ between physicians and nurses. In addition, prior research has clarified decision-making processes and related factors from patients’ perspectives [16,17], contributing valuable insights into patient experiences. Furthermore, although models of SDM-based decision-making have been developed [18], their effective implementation in clinical practice remains insufficiently examined. Taken together, these findings suggest that a comprehensive understanding of SDM facilitators—particularly from a comparative and interprofessional perspective—remains lacking.

SDM is considered most effective when facilitated by multidisciplinary teams that include not only physicians, but also nurses [19]. Furthermore, multidisciplinary collaboration has been reported to improve renal outcomes in the context of renal replacement therapy decision support [20], underscoring the importance of collaborative SDM practices across professionals. However, it is important to distinguish between multiprofessional involvement and interprofessional collaboration; the latter requires active coordination, communication, and shared responsibility among professionals, reflecting a more integrated and collaborative approach to decision-making. Therefore, understanding differences in facilitators of SDM between physicians and nurses within such collaborative contexts is essential for promoting its implementation in practice.

Therefore, the aim of this study was to compare factors that facilitate shared decision-making in renal replacement therapy between physicians and nurses using text mining analysis, and to identify implications for improving interprofessional clinical practice.

## 2. Materials and Methods

### 2.1. Survey Participants

Requests for research cooperation were mailed to the directors of the 442 facilities (excluding clinics) out of the 523 nationwide insurance medical institutions that had submitted notifications for renal replacement therapy management fees to the regional welfare bureaus. The participants comprised 549 physicians and nurses (250 physicians and 299 nurses) affiliated with nephrology departments at 93 facilities that agreed to participate in the study. Physicians and nurses were selected as study participants because they play central roles in decision-making support for renal replacement therapy and are directly involved in SDM processes in clinical practice.

Physicians and nurses with less than one year of experience in nephrology were excluded, as their primary focus is typically on adapting to the workplace environment and acquiring basic clinical skills. Physicians and nurses who had not been involved in renal replacement therapy selection support within the previous four years were also excluded.

### 2.2. Data Collection

A request letter containing a QR code was distributed to eligible physicians and nurses through the directors of each participating facility. We conducted a self-administered web-based questionnaire survey, which was commissioned to an external survey company under the supervision of the research team. The survey was conducted between December 2024 and March 2025. The average time required to complete the questionnaire was approximately 15 min.

### 2.3. Survey Content

The survey comprised seven items: age, sex, occupation, years of experience as a physician or nurse, years of experience in nephrology, years of experience in renal replacement therapy selection support, and possession of a nephrology specialist or certified nurse specialist. In addition, participants were asked about the presence or absence of barriers to SDM. For respondents who reported the presence of barriers, multiple applicable items were selected from a list of 13 potential barriers to SDM practice, developed based on prior research [21,22] (Supplementary Material S1 (1)(2)).

Finally, participants were asked the following open-ended question: “What do you think is necessary to facilitate SDM in renal replacement therapy decision support?” (Supplementary Material S1 (3)) Quantitative survey and free-response data obtained from this question were used in the analyses.

### 2.4. Analysis Methods

Descriptive statistics were calculated as mean ± standard deviation for age, years of experience, years of experience in nephrology, and years of experience in renal replacement therapy selection support. Frequencies and percentages were calculated for sex, possession of specialized qualifications, and the presence and content of barriers to SDM practice. The Mann–Whitney U test and chi-square test were used to compare physicians and nurses.

To identify profession-specific factors facilitating SDM, quantitative text analysis was conducted using free-response data. This method was adopted because it quantitatively organizes word occurrence in textual data, allowing objective and comparable identification of differences in perceived SDM facilitation factors between physicians and nurses. KH Coder 3.Beta.03i (Ritsumeikan University, Kyoto, Japan) [23] was used for analysis. KH Coder extracts morphemes, the smallest meaningful units of language, from textual data and enables statistical analysis, thereby facilitating the identification of overall trends while minimizing researcher bias.

Quantitative text analysis was conducted in accordance with the procedures described in the KH Coder Manual. As a preprocessing step, morphological analysis was performed, and the extracted word list was reviewed. Compound terms that were considered appropriate to be treated as single units—such as renal replacement therapy, decision-making, renal function, kidney failure, kidney transplantation, hemodialysis, peritoneal dialysis, nurse, values, healthcare professionals, information provision, multidisciplinary, necessity, level of understanding, and trust relationship—were defined as forced extraction terms.

In addition, commonly used words that were deemed unnecessary for analysis, such as think, were specified as deleted words. Synonymous terms were unified using replacement settings; for example, renal replacement therapy and RRT, chronic kidney disease and CKD, and peritoneal dialysis and PD were treated as identical terms. Subsequent analyses, including correspondence analysis and co-occurrence network analysis, were conducted as described below.

The total number of extracted words, number of words used in the analysis, and number of sentences were calculated separately for physicians and nurses. To identify the characteristic terms related to SDM promotion factors by profession, a correspondence analysis was performed using profession (physician or nurse) as an external variable. Correspondence analysis enables the identification and visualization of characteristic terms based on differences between groups [23]. The results are displayed on a one-dimensional plot with the origin set at zero. Words located near the origin were commonly used by both professionals, whereas words located farther from the origin were used more frequently by physicians and nurses.

Finally, a co-occurrence network analysis was conducted to examine the strong co-occurrence relationships among words and identify themes related to SDM promotion factors by profession. Co-occurrence networks were constructed separately for physicians and nurses. In the co-occurrence network analysis, the extracted diagram shows that words with strong associations form clusters, which are presented as subgraphs representing groups of related terms. When naming each subgraph, the context in which the words were used in the original text was carefully examined, and validity was ensured through supervision by a renal care expert and a qualitative researcher who were co-investigators. Quotation marks (“ ”) indicate extracted words, angle brackets (⟨ ⟩) indicate free-text responses.

Statistical analyses were performed using SPSS version 29 with the level of significance set at 5%.

### 2.5. Ethical Considerations

This study was approved by the Ethics Review Committee of Nagoya City University. The survey was conducted online, and participants were provided information regarding the study objectives, significance, methods, and ethical considerations as well as assurance of anonymity. Participation was voluntary, and informed consent was obtained when participants indicated their agreement by selecting a checkbox on the web-based survey form. In addition, a business outsourcing agreement concerning the protection of personal information and privacy was established with a web survey company, and all survey data were received and stored in password-protected files.

## 3. Results

### 3.1. Participant Characteristics

Responses were obtained from 238 participants (43.4%). There were 107 physicians (42.8%) and 131 nurses (43.8%). Among these, four physicians and nine nurses with no experience guiding patients in selecting renal replacement therapy within the past four years were excluded. The final survey population comprised 103 physicians (valid response rate: 41.2%) and 122 nurses (valid response rate: 44.8%). All respondents provided answers to the open-ended questions. The mean age of physicians was 45.9 ± 8.9 years, and that of nurses was 46.6 ± 7.8 years. There were 85 male physicians, 18 female physicians, 16 male nurses and 106 female nurses. (Table 1).

### 3.2. Presence and Types of Barriers to SDM Practice

Overall, 144 respondents (64.0%) reported barriers to implementing SDM practices. The most frequently reported barrier was the inability to secure sufficient time (103 respondents, 71.5%), followed by insufficient understanding among patients and families (80 respondents, 55.6%).

A significantly higher proportion of physicians reported barriers to SDM practice than nurses (physicians: 75/103, 72.8%; nurses: 69/122, 56.6%; *p* = 0.011). Regarding specific barriers, the inability to secure sufficient time was reported more frequently by physicians (60/103, 58.3%) than nurses (43/122, 35.2%; *p* = 0.019). In contrast, nurses reported a lack of knowledge regarding SDM methods or procedures (16/69, 23.2%) more frequently than physicians (7/75, 9.3%; *p* = 0.023). Nurses also reported difficulty in obtaining physician cooperation (12/69, 17.4% vs. 5/75, 6.7%; *p* = 0.046) (Table 2).

### 3.3. Results of Quantitative Text Analysis

Morphological analysis was performed using KH Coder 3. Beta.03i yielded 2,810 words extracted from physicians and 4206 words from nurses. Of these, 1312 words from physicians and 1856 words from nurses were used in the analysis (Table 3).

#### 3.3.1. Results of Correspondence Analysis

**Figure 1:**
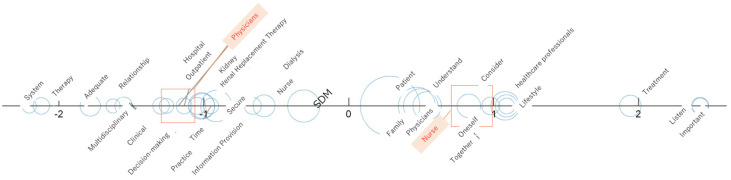
Plot of Correspondence analysis.

Correspondence analysis identified words characteristic of each profession. For physicians words including “system,” “therapy,” “adequate,” “multidisciplinary,” and “relationship” were identified. For nurses, words including “important,” “listen,” “treatment,” “lifestyle,” “healthcare professionals,” and “consider” were identified (Figure 1).

The results of the correspondence analysis using physicians and nurses as external variables were visualized and are presented in Figure 1. Words located near the origin were commonly used by both professionals, whereas words located farther from the origin were used more frequently by physicians and nurses. Circles represent individual words, and their size reflects word frequency, with larger circles indicating higher frequency. Square size represents the relative amount of data for each professional group. The axis shows the primary contrast, with positive and negative values indicating opposing tendencies between groups. Category points were moved toward the center while preserving relative positions.

#### 3.3.2. Co-Occurrence Network Analysis

As a result of the co-occurrence network analysis, six subgraphs were identified for both physicians and nurses. These subgraphs were constructed based on co-occurrence relationships among words [23]. When naming each subgraph, the context in which the words were used in the original text was carefully examined in accordance with the approach described in Reference [23].

**Figure 2:**
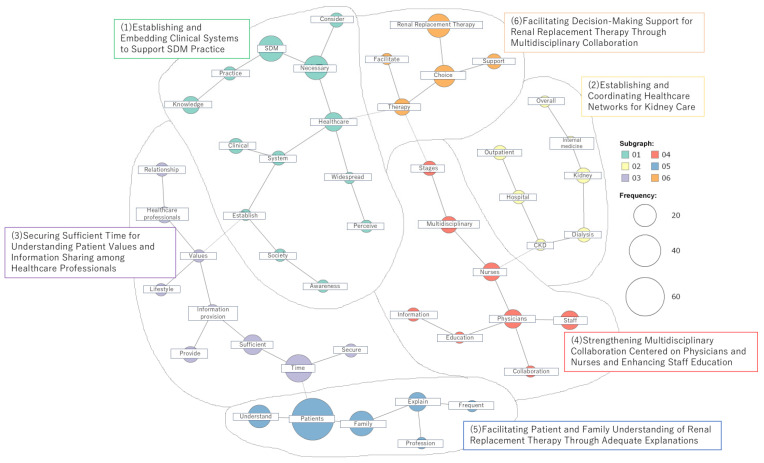
Factors facilitating physicians’ implementation of shared decision-making. Numbers (01–06) indicate individual categories.

Factors facilitating physicians’ implementation of shared decision-making are shown in Figure 2.

1. Establishing and Embedding Clinical Systems to Support SDM Practice

A co-occurrence network centered on terms such as “necessary,” “SDM,” “practice,” “knowledge,” “healthcare,” “widespread,” “system,” “clinical,” and “establish” included the following free-response contents: ⟨Clinical support by staff knowledgeable and skilled in SDM⟩, ⟨Regional healthcare systems capable of addressing all treatment preferences⟩, ⟨Sufficient consultation periods and time⟩, ⟨a healthcare system enabling repeated discussions⟩, ⟨society-wide awareness of SDM⟩, ⟨promoting SDM. Raising patient awareness of the SDM concept⟩, ⟨disseminating the SDM concept⟩, ⟨ensuring all healthcare staff understand and embrace it⟩. This network was named “Establishing and Embedding Clinical Systems to Support SDM Practice.”

2. Establishing and Coordinating Healthcare Networks for Kidney Care

A co-occurrence network centered on terms such as “dialysis,” “kidney,” “internal medicine,” “overall,” “CKD,” “hospital,” and “outpatient” included the following free-response contents: ⟨early consultation with nephrology specialists⟩, ⟨an increase in hospitals providing renal replacement therapy selection clinics, as well as an increase in hospitals referring patients to these clinics⟩, ⟨nationwide standardization of support content⟩, ⟨small- and medium-sized hospitals, home-visit medical services, and institutions providing end-of-life care envisioned as receiving facilities⟩, ⟨to promote SDM, a foundation enabling the selection of all treatment options, including collaboration among medical institutions, must be established⟩. This network was named “Establishing and Coordinating Healthcare Networks for Kidney Care.”

3. Securing Sufficient Time for Understanding Patient Values and Information Sharing among Healthcare Professionals

A co-occurrence network centered on terms such as “time,” “sufficient,” “information provision,” “values,” “healthcare professionals,” “lifestyle,” and “secure” included the following free-response contents: ⟨securing sufficient time to provide information to patients and allowing them time to understand and process their emotions⟩, ⟨securing sufficient time for explanations to patients and their families, including experiences that help them visualize concrete scenarios⟩, ⟨healthcare professionals understanding patients’ and families’ living situations and values, ideally with sufficient time for multidisciplinary discussion⟩. This network was named “Securing Adequate Time to Understand Patient Values and Information Sharing among Healthcare Professionals.”

4. Strengthening Multidisciplinary Collaboration Centered on Physicians and Nurses and Enhancing Staff Education

A co-occurrence network centered on terms such as “physicians,” “nurses,” “multidisciplinary,” “staff,” “collaboration,” “education,” and “information” included the following free-response contents:⟨the need for collaboration among multiple professions, including nurses, pharmacists, and medical social workers, to promote SDM⟩, ⟨collaboration between physicians and nurses⟩, ⟨enhancement of patient education tools and improvement of healthcare professionals’ communication skills to support patient-centered care⟩, ⟨adequate manpower and staff education⟩, ⟨ensuring that all staff involved possess sufficient knowledge related to renal replacement therapy decision support⟩. This network was named “Strengthening Multidisciplinary Collaboration Centered on Physicians and Nurses and Enhancing Staff Education.”

5. Facilitating Patient and Family Understanding of Renal Replacement Therapy Through Adequate Explanations

A co-occurrence network centered on terms such as “patients,” “family,” “explain,” “frequent,” “profession,” and “understand” included the following free-response contents: ⟨Explaining all renal replacement therapy options to find the best approach⟩, ⟨Providing patients with sufficient information. Strive to offer explanations that support not only the patient but also their family⟩, ⟨Patients need education on shared decision-making. Their right to self-determination must be fully guaranteed⟩, ⟨Patients must possess sufficient literacy to make self-determined decisions⟩, ⟨Patients and families must understand their condition. They must grasp the general advantages and disadvantages of each renal replacement therapy⟩, ⟨Patients should be able to evaluate the benefits and drawbacks of each renal replacement therapy in relation to their own situation. They should be able to assess whether each therapy is feasible and achievable given their specific condition⟩, ⟨Not only healthcare providers’ understanding, but also the patient’s understanding⟩ ⟨Involving not only the patient but also family members⟩. This network was named “Facilitating Patient and Family Understanding of Renal Replacement Therapy Through Adequate Explanations.”

6. Facilitating Decision-Making Support for Renal Replacement Therapy Through Multidisciplinary Collaboration

A co-occurrence network centered on terms such as “Renal Replacement Therapy,” “choice,” “therapy,” “facilitate,” and “support” included the following free-response contents: ⟨Building trust with patients, assessing their awareness, values, and judgment regarding therapy selection—including those of their caregivers—and advancing therapy selection support through multidisciplinary collaboration in a way that is individually appropriate and not overly burdensome⟩, ⟨Starting from chronic phase management, healthcare providers continuously engage with patients. Patients should feel assured that they will be thoroughly cared for regardless of the choice made⟩, ⟨When a trusting relationship with the patient is established, SDM practice during renal replacement therapy selection is likely to proceed smoothly⟩. This network was named “Facilitating Decision-Making Support for Renal Replacement Therapy through Multidisciplinary Collaboration.”

**Figure 3:**
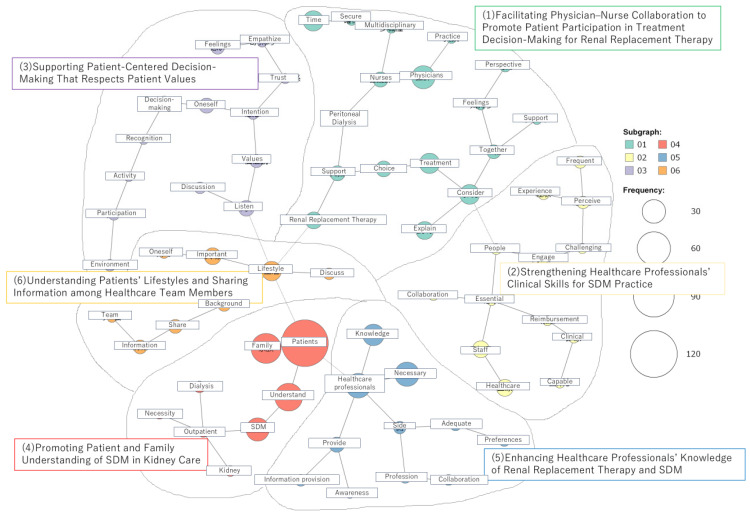
Factors facilitating nurses’ implementation of shared decision-making. Numbers (01–06) indicate individual categories.

Factors facilitating nurses’ implementation of shared decision-making are shown in Figure 3.

1. Facilitating Physician–Nurse Collaboration to Promote Patient Participation in Treatment Decision-Making for Renal Replacement Therapy

A co-occurrence network centered on terms such as “physicians,” “nurses,” “multidisciplinary,” “secure,” “time,” “treatment,” “consider,” “together,” “feelings,” “support,” and “Renal Replacement Therapy” included the following free-response contents: ⟨Physicians and nurses should share information on equal footing through conferences and similar forums⟩, ⟨Nurses want to support patients’ thoughts, lifestyles, and daily lives. Physicians want to provide safe medical care. A willingness to meet each other halfway is crucial⟩, ⟨Shared awareness among healthcare providers, family, and the patient themselves to provide information fairly and help them choose the best treatment for that patient⟩, ⟨A stance of eliciting what gives meaning to life and what is cherished, then considering together with the patient a future way of living that is true to who they are⟩. This network was named “Facilitating Physician–Nurse Collaboration to Promote Patient Participation in Treatment Decision-Making for Renal Replacement Therapy.”

2. Strengthening Healthcare Professionals’ Clinical Skills for SDM Practice

A co-occurrence network centered on terms such as “healthcare,” “staff,” “essential,” “people,” “engage,” “challenging,” “perceive,” “experience,” “reimbursement,” “clinical,” and “collaboration” included the following free-response contents: ⟨staff participation in learning sessions, case presentations, and case review meetings to deepen understanding of SDM and share information among staff⟩, ⟨deepening knowledge of each treatment option and accumulating clinical experience⟩, ⟨the need for healthcare professionals to continuously acquire knowledge and skills and engage in ongoing self-improvement⟩, ⟨the perception that sufficient skills on the part of healthcare staff are essential⟩. This network was named “Strengthening Healthcare Professionals’ Clinical Skills for SDM Practice.”

3. Supporting Patient-Centered Decision-Making That Respects Patient Values

A co-occurrence network centered on terms such as “values,” “listen,” “trust,” “empathize,” “decision-making,” “feelings,” “oneself,” “recognition,” “activity,” and “participation,” included the following free-response contents: ⟨Providing the best possible care as a healthcare professional and aligning with the patient’s wishes and values⟩, ⟨I believe understanding the patient first is crucial. To do this, listening to their life background, values, and feelings is paramount, and active listening is essential⟩, ⟨drawing out the patient’s values and outlook on life⟩, ⟨I believe it is necessary to promote patient-centered decision-making while considering together whether it holds meaning for the patient’s life⟩, ⟨It is also necessary to ensure patients firmly recognize that decision-making ultimately rests with them⟩, ⟨Providing easily understandable explanations of necessary knowledge and information, allowing patients and families to experience actual treatments, and fostering information exchange with patients are crucial for enabling them to make decisions based on their own will⟩. This network was named “Supporting Patient-Centered Decision-Making That Respects Patient Values.”

4. Promoting Patient and Family Understanding of SDM in Kidney Care

A co-occurrence network centered on terms such as “patients,” “family,” “understand,” “SDM,” “outpatient,” and “necessity,” included the following free-response contents: ⟨First and foremost, healthcare providers must explain the concept of SDM to patients and their families and ensure they understand it⟩, ⟨Patients and their families involved in the patient’s care should develop an interest in and understanding of SDM. Increasing general public awareness of SDM⟩. This network was named “Promoting Patient and Family Understanding of SDM in Kidney Care.”

5. Enhancing Healthcare Professionals’ Knowledge of Renal Replacement Therapy and SDM

A co-occurrence network centered on terms such as “healthcare professionals,” “knowledge,” “necessary,” “provide,” “information provision,” “profession,” and “collaboration,” included the following free-response contents: ⟨SDM is difficult without a certain level of knowledge. Therefore, involved healthcare professionals need to gain knowledge through study sessions, etc.⟩, ⟨Improving healthcare professionals’ knowledge and awareness is important⟩, ⟨We believe healthcare professionals need sufficient knowledge when advising on treatment choices⟩, ⟨We believe involved healthcare professionals need to gain knowledge through study sessions⟩, ⟨Due to individual differences in guidance based on healthcare professionals’ knowledge and experience, we feel overall knowledge needs to be raised⟩. This network was named “Enhancing Healthcare Professionals’ Knowledge of Renal Replacement Therapy and SDM.”

6. Understanding Patients’ Lifestyles and Sharing Information among Healthcare Team Members

A co-occurrence network centered on terms such as “lifestyle,” “discuss,” “important,” “oneself,” “background,” “share,” “information,” and “team” included the following free-response contents: ⟨listening carefully to patients’ life backgrounds, values, and feelings as a top priority⟩, ⟨thoroughly understanding patients’ living situations, work, hobbies, level of understanding, emotional state, and family environment⟩, ⟨eliciting patients’ sense of purpose and life background and sharing this information among healthcare professionals to better support patients⟩, ⟨drawing out the thoughts of patients and their families and integrating renal replacement therapy into their daily lives⟩, ⟨deepening relationships with patients and their families to understand individual life backgrounds and perspectives⟩, ⟨listening to patients’ life backgrounds and values and sharing this information with families while supporting joint decision-making⟩. This network was named “Understanding Patients’ Lifestyles and Sharing Information among Healthcare Team Members.”

In summary, six networks were identified by both physicians and nurses (Table 4).

## 4. Discussion

### 4.1. Barriers to SDM Practice in Supporting Renal Replacement Therapy Selection

Approximately 60% of the participants reported experiencing barriers to SDM practice, with physicians perceiving such barriers significantly more frequently than nurses. The most commonly reported barrier was the inability to secure sufficient time (approximately 70%). Profession-specific analysis further demonstrated that physicians reported difficulty in securing sufficient time significantly more often than nurses. This finding is consistent with those of previous studies [22], suggesting that physicians experience challenges in allocating adequate time for the dialog necessary to implement SDM within routine clinical consultations. This tendency may reflect the structural and organizational characteristics of physicians’ roles, which often involve time-constrained decision-making responsibilities, high patient volumes, and the need to manage complex clinical information within limited consultation periods.

The second most frequently cited barrier was insufficient understanding among the patients and their families (approximately 50%). One of the four essential elements of SDM is the involvement of both healthcare providers and patients in the decision-making process [7]. Moreover, SDM requires not only healthcare providers, but also patients, to actively engage in decision-making through behavioral changes [21]. The present findings suggest that healthcare professionals should recognize the importance of disseminating the concept of SDM to patients and their families to facilitate their active participation in treatment decisions.

In contrast, nurses more frequently reported barriers, such as lack of knowledge regarding SDM methods or procedures and difficulty in obtaining physician cooperation, compared to physicians. These findings indicate that nurses may experience challenges related to the practical implementation of SDM and interprofessional collaboration in decision-making processes. This may reflect the fact that nurses, who are closely involved in patient support and communication, are more likely to recognize the need for practical knowledge and skills to facilitate SDM in clinical settings. In other words, their perception of knowledge gaps may arise not from a lack of educational opportunities per se, but from the increasing demands of supporting SDM in everyday practice. This highlights the need for clear guidance on SDM practices and strengthened collaborative structures between physicians and nurses to support effective SDM implementation.

Taken together, these findings suggest that physicians tend to perceive structural constraints, whereas nurses are more sensitive to practice-based and relational challenges in implementing SDM.

### 4.2. Factors Facilitating SDM in Renal Replacement Therapy Choice Support

Based on the results of correspondence and co-occurrence network analyses of free-response data from physicians and nurses, factors facilitating SDM in renal replacement therapy choice support were identified. In this section, these factors are discussed by distinguishing those commonly recognized by both physicians and nurses from those characteristic of each profession.

#### 4.2.1. Common Facilitators of Shared Decision-Making Among Physicians and Nurses

Two facilitators of SDM that were common to both physicians and nurses were identified as patient-related facilitators and clinician-related facilitators.

The first patient-related facilitator promoted the patients’ and families’ understanding of SDM. Both the physicians’ network “Facilitating Patient and Family Understanding of Renal Replacement Therapy Through Adequate Explanations” and the nurses’ network “Promoting Patient and Family Understanding of SDM in Kidney Care” consistently indicated that deepening patients’ and families’ understanding facilitates their active participation in SDM. SDM is a process in which healthcare professionals share their specialized knowledge with patients and their families while patients contribute their values, preferences, and life circumstances, enabling the collaborative determination of an optimal treatment plan [24,25]. Enhancing patients’ and families’ understanding of SDM supports their active engagement in decision-making, promotes value-based treatment choices, and represents a core element in advancing SDM practices.

The second facilitator, common to physicians and nurses, involves clinician-related factors, specifically staff education and multidisciplinary collaboration. With regard to staff education, the physicians’ network “Strengthening Multidisciplinary Collaboration Centered on Physicians and Nurses and Enhancing Staff Education” and the nurses’ networks “Strengthening Healthcare Professionals’ Clinical Skills for SDM Practice” and “Enhancing Healthcare Professionals’ Knowledge of Renal Replacement Therapy and SDM” indicated that strengthening educational systems aimed at improving healthcare professionals’ knowledge and practical skills is essential for promoting SDM. These findings suggest that SDM should be positioned not as a practice dependent on individual healthcare professionals’ experience or competence but as an organizational educational framework that includes structured training programs through development of standardized manuals. This approach may ensure a consistent level of SDM practice and reduce variations in the quality of decision support among healthcare professionals.

Regarding multidisciplinary collaboration, physicians emphasized networks such as “Facilitating Decision-Making Support for Renal Replacement Therapy Through Multidisciplinary Collaboration” and “Strengthening Multidisciplinary Collaboration Centered on Physicians and Nurses and Enhancing Staff Education”, whereas nurses emphasized the network “Facilitating Physician–Nurse Collaboration to Promote Patient Participation in Treatment Decision-Making for Renal Replacement Therapy”, underscoring the particular importance of physician–nurse collaboration. Previous studies have reported that collaboration between physicians and nurses contributes to improved quality of care [26] and enhanced teamwork [27]. Accordingly, physician–nurse collaboration is recognized as a fundamental component of high-quality care and is also considered a crucial element in effective SDM practice.

#### 4.2.2. Physician-Specific Facilitators of Shared Decision-Making

Physician-specific facilitators of SDM were identified in “Establishing and Embedding Clinical Systems to Support SDM Practice”, “Establishing and Coordinating Healthcare Networks for Kidney Care”, and “Securing Sufficient Time for Understanding Patient Values and Information Sharing among Healthcare Professionals”. These findings suggest that physicians primarily recognize the facilitators of SDM through structural and environmental perspectives centered on the establishment and improvement of healthcare systems.

The correspondence analysis further identified terms such as “system,” “therapy,” “adequacy,” “relationship,” and “multidisciplinary” as characteristic words among physicians. This indicates that physicians perceive SDM not merely as a practical challenge within individual clinical encounters but as a process that is advanced through the development of institutional, organizational, and regional healthcare systems.

In particular, physicians emphasized regional and interfacility collaboration, as reflected in the network “Establishing and Coordinating Healthcare Networks for Kidney Care.” Decision support for renal replacement therapy requires long-term and continuous engagement, from the conservative management phase to the initiation of dialysis or kidney transplantation. Consequently, SDM support limited to a single healthcare institution has inherent limitations, and the importance of inter-institutional collaboration and information-sharing mechanisms has been highlighted in previous research [28]. Against this background, physicians likely perceive the establishment of coordinated healthcare networks as key facilitators of SDM.

Furthermore, the network “Securing Sufficient Time for Understanding Patient Values and Information Sharing among Healthcare Professionals” reflects physicians’ recognition that current clinical systems often lack sufficient time resources, despite SDM being fundamentally premised on thorough dialog and deliberation. In addition, the network “Establishing and Embedding Clinical Systems to Support SDM Practice” suggests that physicians view SDM not as a practice dependent on individual healthcare providers’ efforts but as one requiring institutional frameworks, including clinical systems, reimbursement structures, and regional healthcare infrastructures.

Overall, physicians recognized the facilitators of SDM primarily in terms of structural and environmental dimensions, emphasizing the importance of healthcare system development, inter-institutional collaboration, and securing adequate time and organizational resources.

#### 4.2.3. Nurse-Specific Facilitators of Shared Decision-Making

Nurse-specific facilitators of SDM were identified in the networks “Supporting Patient-Centered Decision-Making That Respects Patient Values” and “Understanding Patients’ Lifestyles and Sharing Information among Healthcare Team Members.” These findings indicate that nurses tend to recognize facilitators of SDM that emphasize practical and interpersonal aspects, such as understanding patients’ values and lifestyles, and engaging in collaborative information sharing with other healthcare professionals.

Correspondence analysis further revealed characteristic terms among nurses, including “important,” “listen,” “treatment,” “lifestyle,” “together,” and “consider.” These terms likely reflect the fundamental nursing perspective of empathizing with patients’ experiences and emotions while attending to their psychological and social contexts.

Respect for patient values, as represented by the network “Supporting Patient-Centered Decision-Making That Respects Patient Values,” was identified as a core element of SDM practice, emphasizing the careful elicitation of patients’ narratives and incorporation of their perspectives into the decision-making process. Additionally, the network “Understanding Patients’ Lifestyles and Sharing Information among Healthcare Team Members” highlights the importance of comprehensive patient understanding that extends beyond medical information alone. This perspective is particularly relevant in renal replacement therapy selection because treatment decisions substantially affect patients’ daily lives, family relationships, employment, and social participation.

In summary, nurses recognized facilitators of SDM that emphasize individualized, practical, and interpersonal approaches, including supporting patient-centered decision-making through multidisciplinary collaboration and information sharing aligned with patients’ values and life circumstances.

From a practical perspective, the findings of this study suggest the need for tailored strategies to promote SDM implementation across professional roles. For physicians, interventions addressing structural constraints—such as optimizing clinical workflows and ensuring sufficient time for patient dialog—may be particularly important. For nurses, providing practical guidance and skill-based training on SDM methods and communication strategies may help address perceived knowledge needs arising from everyday clinical practice. In addition, the development of structured protocols and the strengthening of interprofessional collaboration frameworks may facilitate the systematic integration of SDM into routine renal care.

Thus, the factors facilitating shared decision-making in renal replacement therapy selection demonstrate both commonalities and profession-specific differences, underscoring the importance of addressing these factors to advance SDM in clinical practice.

### 4.3. Limitations of This Study and Future Directions

This study has several limitations. First, the response rate was 43.4%, and participation was voluntary. Therefore, selection bias cannot be excluded, as healthcare professionals who are more interested in SDM may have been more likely to respond. Consequently, the findings may overrepresent positive attitudes toward SDM and may not fully reflect the perceptions of all physicians and nurses involved in renal replacement therapy decision support. Overall, these factors may limit the representativeness of the sample and the generalizability of the findings.

Second, data were collected using a web-based questionnaire, which may have excluded healthcare professionals with limited access to or familiarity with digital technologies. This may have introduced additional selection bias.

Third, the study relied on self-reported perceptions of facilitators and barriers to SDM rather than direct observation of clinical practice. Thus, the identified factors represent participants’ subjective recognition and do not necessarily indicate that these factors objectively improve SDM implementation or patient outcomes.

Fourth, although quantitative text analysis using KH Coder enabled objective and systematic analysis of free-text responses, the interpretation and labeling of co-occurrence networks inevitably involved researchers’ judgment. While efforts were made to minimize bias through transparent analytic procedures, some degree of interpretive subjectivity cannot be completely eliminated.

Fifth, this study included physicians and nurses working in facilities that had submitted notifications for renal replacement therapy management fees in Japan. Therefore, the findings may not be generalizable to other healthcare systems, smaller clinics, or countries with different institutional frameworks for renal care. In particular, differences in healthcare policies, reimbursement systems, and cultural contexts may influence the implementation and perception of SDM, which should be considered when interpreting the findings.

Sixth, this study focused exclusively on healthcare professionals’ perspectives. Patients’ and families’ perceptions of facilitators of SDM were not examined. Future research should incorporate patient perspectives and mixed-method approaches to further validate and refine the facilitators identified in this study.

Seventh, this study did not examine regional or institutional differences, such as variations between large and small hospitals or across different geographic areas. These contextual factors may influence the implementation of SDM and the perceived facilitators and barriers. Therefore, future research should consider incorporating such comparisons to provide a more comprehensive understanding.

Finally, the findings of this study represent the factors that healthcare providers recognize as facilitating SDM. However, this study does not examine whether these factors facilitate SDM in practice. Future studies should employ different methodological approaches to verify whether the factors identified in this study facilitate SDM.

## 5. Conclusions

In renal replacement therapy selection support, the facilitators of SDM commonly recognized by both physicians and nurses were the promotion of SDM understanding among patients and their families and staff education with interprofessional collaboration. In terms of profession-specific facilitators, physicians tend to emphasize the structural and environmental aspects of healthcare systems, such as inter-institutional collaboration frameworks and the availability of appropriate clinical environments and time resources. In contrast, nurses emphasized practical and interpersonal aspects, including understanding patients’ values and life backgrounds and collaborating with other professionals through information sharing. These findings indicate that facilitators of SDM in renal replacement therapy selection support include both commonalities and differences between professions, suggesting the importance of focusing on these factors to facilitate SDM in clinical practice.

From a practical perspective, these findings suggest the need for developing structured SDM support systems, including multidisciplinary training programs, standardized protocols, and organizational frameworks that enable effective collaboration and sufficient time for patient engagement. Furthermore, future research should examine the effectiveness of these strategies through longitudinal and intervention-based studies.

Previous research on healthcare professionals’ involvement in SDM has primarily focused on physicians, with limited attention given to nurses. The findings of this study suggest the importance of interprofessional collaboration and indicate that profession-specific factors facilitating SDM may provide valuable insights not only for Japan but also for other healthcare systems facing challenges in patient-centered care and interprofessional collaboration.

## Figures and Tables

**Table 1:** Demographic Characteristics of Participants (n = 225).

		Overall (n = 225)		Physicians (n = 103)		Nurses (n = 122)		*p*
Age ^(1)^		46.3 ± 8.3		45.9 ± 8.9		46.6 ± 7.8		0.349
Gender ^(2)^	Male	101	(44.9)	85	(82.5)	16	(13.1)	<0.001 **
	Female	124	(55.1)	18	(17.5)	106	(86.9)	
Years of Experience ^(1)^			21.4 ± 8.7		19.6 ± 9.0		23.0 ± 8.1	0.001 **
	<5	3	(1.3)	2	(1.9)	1	(0.8)	
	5~<10	16	(7.1)	12	(11.7)	4	(3.3)	
	10~<15	32	(14.2)	18	(17.5)	14	(11.5)	
	15~<20	41	(18.2)	21	(20.4)	20	(16.4)	
	≥20	133	(59.1)	50	(48.5)	83	(68.0)	
Years of Nephrology Experience ^(1)^			14.4 ± 8.6		16.5 ± 9.0		12.6 ± 7.9	0.001 **
	<5	25	(11.1)	10	(9.7)	15	(12.3)	
	5~<10	49	(21.8)	15	(14.6)	34	(27.9)	
	10~<15	44	(19.6)	19	(18.4)	25	(20.5)	
	15~<20	47	(20.9)	23	(22.3)	24	(19.7)	
	≥20	60	(26.7)	36	(35.0)	24	(19.7)	
Years of experience supporting renal replacement therapy ^(1)^			9.4 ± 7.7		13.1 ± 8.7		6.2 ± 5.0	<0.001 **
	<5	80	(35.6)	19	(18.4)	61	(50.0)	
	5~<10	43	(19.1)	17	(16.5)	26	(21.3)	
	10~<15	52	(23.1)	28	(27.2)	24	(19.7)	
	15~<20	25	(11.1)	16	(15.5)	9	(7.4)	
	≥20	25	(11.1)	23	(22.3)	2	(1.6)	
Specialist physician or certified nursing qualification (CNS or CN) ^(2)^	Yes	135	(60.0)	85	(82.5)	50	(41.0)	<0.001 **
	No	90	(40.0)	18	(17.5)	72	(59.0)	

^(1)^ Mann–Whitney U test ^(2)^ Chi-square test ** *p* < 0.01. Values are presented as number (%) or mean ± standard deviation. Abbreviations: CNS, Certified Nurse Specialist; CN, Certified Nurse. Certified Nurse Specialist and Certified Nurse are certifications granted by the Japanese Nursing Association.

**Table 2:** Distribution of Barriers to SDM Practice (n = 225).

**<Are there barriers to implementing SDM?> ^(1)^**								
		**All participants (*n* = 225)**		**Physicians (*n* = 103)**		**Nurses (*n* = 122)**		***p***
	Yes	144	(64.0)	75	(72.8)	69	(56.6)	0.011 *
	No	81	(36.0)	28	(27.2)	53	(43.4)	
**When barriers exist <What are the barriers to SDM?> ^(1)^**								
		**Participants reporting barriers (*n* = 144)**		**Physicians (*n* = 75)**		**Nurses (*n* = 69)**		***p***
Inability to secure sufficient time	Yes	103	(71.5)	60	(88.0)	43	(62.3)	0.019 *
	No	41	(28.5)	15	(12.0)	26	(37.7)	
Inability to secure an appropriate location	Yes	43	(29.9)	19	(25.3)	24	(34.8)	0.216
	No	101	(70.1)	56	(74.7)	45	(65.2)	
Lack of knowledge regarding methods or procedure	Yes	23	(16.0)	7	(9.3)	16	(23.2)	0.023 *
	No	121	(84.0)	68	(90.7)	53	(76.8)	
Perceived lack of necessity	Yes	6	(4.2)	1	(1.3)	5	(7.2)	0.076
	No	138	(95.8)	74	(98.7)	64	(92.8)	
Lack of understanding among physicians	Yes	13	(9.0)	4	(5.3)	9	(13.0)	0.107
	No	131	(91.0)	71	(94.7)	60	(87.0)	
Inability to obtain physician cooperation	Yes	17	(11.8)	5	(6.7)	12	(17.4)	0.046 *
	No	127	(88.2)	70	(93.3)	57	(82.6)	
Lack of understanding among nurses	Yes	12	(8.3)	3	(4.0)	9	(13.0)	0.050
	No	132	(91.7)	72	(96.0)	60	(87.0)	
Inability to obtain nurse cooperation	Yes	10	(6.9)	5	(6.7)	5	(7.2)	0.891
	No	134	(93.1)	70	(93.3)	64	(92.8)	
Lack of interprofessional cooperation	Yes	29	(20.1)	12	(16.0)	17	(24.6)	0.197
	No	115	(79.9)	63	(84.0)	52	(75.4)	
Insufficient acceptance among healthcare providers	Yes	45	(31.2)	21	(28.0)	24	(34.8)	0.380
	No	99	(68.8)	54	(72.0)	45	(65.2)	
Insufficient understanding among patients and families	Yes	80	(55.6)	41	(54.7)	39	(56.5)	0.823
	No	64	(44.4)	34	(45.3)	30	(43.5)	
Inability to obtain patient and family cooperation	Yes	40	(27.8)	22	(29.3)	8	(26.1)	0.664
	No	104	(72.2)	53	(70.7)	51	(73.9)	
Perceived lack of necessity among patients and families	Yes	48	(33.3)	24	(32.0)	24	(34.8)	0.723
	No	96	(66.7)	51	(68.0)	45	(65.2)	

^(1)^ Chi-square test * *p* < 0.05 n (%).

**Table 3:** Number of extracted words by physicians and nurses.

	Physicians	Nurses
Total Extracted Words	2810	4206
Of which used in analysis	1312	1856
Number of sentences	146	209

**Table 4:** Co-occurrence network of physicians and nurses.

<Common Themes Identified in Physicians and Nurses>	
**Physician**	**Nurse**
Facilitating Patient and Family Understanding of Renal Replacement Therapy Through Adequate Explanations (5)	Promoting Patient and Family Understanding of SDM in Kidney Care (4)
Strengthening Multidisciplinary Collaboration Centered on Physicians and Nurses and Enhancing Staff Education (4)	Strengthening Healthcare Professionals’ Clinical Skills for SDM Practice (2)
	Enhancing Healthcare Professionals’ Knowledge of Renal Replacement Therapy and SDM (5)
Facilitating Decision-Making Support for Renal Replacement Therapy Through Multidisciplinary Collaboration (6)	Facilitating Physician–Nurse Collaboration to Promote Patient Participation in Treatment Decision-Making for Renal Replacement Therapy (1)
(Strengthening Multidisciplinary Collaboration Centered on Physicians and Nurses and Enhancing Staff Education (4))	
<Different Themes Identified between Physicians and Nurses>	
**Physicians**	**Nurses**
Establishing and Embedding Clinical Systems to Support SDM Practice (1)	Supporting Patient-Centered Decision-Making That Respects Patient Values (3)
Establishing and Coordinating Healthcare Networks for Kidney Care (2)	Understanding Patients’ Lifestyles and Sharing Information among Healthcare Team Members (6)
Securing Sufficient Time for Understanding Patient Values and Information Sharing among Healthcare Professionals (3)	

Notes: Numbers (1)–(6) refer to the network numbers.

## Data Availability

The data presented in this study are available on request from the corresponding author. The data are not publicly available due to ethical restrictions.

## Supplementary Materials

The following supporting information can be downloaded at: https://www.mdpi.com/article/10.3390/nursrep16040142/s1, Supplementary Material S1. Questionnaire.

## Public Involvement Statement

No public involvement in any aspect of this research.

## Guidelines and Standards Statement

This manuscript was drafted in accordance with the Strengthening the Reporting of Observational Studies in Epidemiology (STROBE) Statement for Cross-Sectional Studies.
