# Supplementary material for: Shared Decision-Making in the Choice of Renal Replacement Therapy: A Comparative Text Mining Analysis of Physicians and Nurses
Source: Nurs Rep. 2026 Apr 16;16(4):142. doi: 10.3390/nursrep16040142 (PMC13119424; doi:10.3390/nursrep16040142)
Supplement: Supplementary file 1 [file nursrep-16-00142-s001.zip › nursrep-4188687-supplementary.pdf]

Supplementary Material 1. Questionnaire

(1) Are there barriers to implementing SDM?
Yes or No

(2) When barriers exist <What are the barriers to SDM?>

1. Inability to secure sufficient time
   Yes or No
2. Inability to secure an appropriate location
   Yes or No
3. Lack of knowledge regarding methods or procedure
   Yes or No
4. Perceived lack of necessity
   Yes or No
5. Lack of understanding among physicians
   Yes or No
6. Inability to obtain physician cooperation
   Yes or No
7. Lack of understanding among nurses
   Yes or No
8. Inability to obtain nurse cooperation
   Yes or No
9. Lack of interprofessional cooperation
   Yes or No
10. Insufficient acceptance among healthcare providers
    Yes or No
11. Insufficient understanding among patients and families
    Yes or No
12. Inability to obtain patient and family cooperation
    Yes or No
13. Perceived lack of necessity among patients and families
    Yes or No

(3) What do you think is necessary to facilitate SDM in renal replacement therapy decision support?
【Free notes in 800 words or less】
